# Deep-Learning-Based Automated Building Construction Progress Monitoring for Prefabricated Prefinished Volumetric Construction

**DOI:** 10.3390/s24217074

**Published:** 2024-11-02

**Authors:** Wei Png Chua, Chien Chern Cheah

**Affiliations:** School of Electrical & Electronic Engineering, Nanyang Technological University, 50 Nanyang Ave, Singapore 639798, Singapore; weipng001@ntu.edu.sg

**Keywords:** deep learning, computer vision, object detection, progress monitoring

## Abstract

Prefabricated prefinished volumetric construction (PPVC) is a relatively new technique that has recently gained popularity for its ability to improve flexibility in scheduling and resource management. Given the modular nature of PPVC assembly and the large amounts of visual data amassed throughout a construction project today, PPVC building construction progress monitoring can be conducted by quantifying assembled PPVC modules within images or videos. As manually processing high volumes of visual data can be extremely time consuming and tedious, building construction progress monitoring can be automated to be more efficient and reliable. However, the complex nature of construction sites and the presence of nearby infrastructure could occlude or distort visual data. Furthermore, imaging constraints can also result in incomplete visual data. Therefore, it is hard to apply existing purely data-driven object detectors to automate building progress monitoring at construction sites. In this paper, we propose a novel 2D window-based automated visual building construction progress monitoring (WAVBCPM) system to overcome these issues by mimicking human decision making during manual progress monitoring with a primary focus on PPVC building construction. WAVBCPM is segregated into three modules. A detection module first conducts detection of windows on the target building. This is achieved by detecting windows within the input image at two scales by using YOLOv5 as a backbone network for object detection before using a window detection filtering process to omit irrelevant detections from the surrounding areas. Next, a rectification module is developed to account for missing windows in the mid-section and near-ground regions of the constructed building that may be caused by occlusion and poor detection. Lastly, a progress estimation module checks the processed detections for missing or excess information before performing building construction progress estimation. The proposed method is tested on images from actual construction sites, and the experimental results demonstrate that WAVBCPM effectively addresses real-world challenges. By mimicking human inference, it overcomes imperfections in visual data, achieving higher accuracy in progress monitoring compared to purely data-driven object detectors.

## 1. Introduction

Today, visual construction monitoring is a quintessential aspect of the construction industry. It is a crucial process that provides updates on various aspects of infrastructure construction by comparing visual data obtained on site against as-planned data [1]. These updates provide project managers with critical information required for evaluation and decision making [2,3]. With the increasing ease of image and video taking, visual data can come from a broad selection of sources, ranging from a smartphone to a camera mounted on an unmanned aerial vehicle (UAV). As a result, the amount of visual data amassed in an average construction project can run up to the thousands over just a few months [4], which can be extremely tedious and time-consuming to analyse manually. In recent years, deep learning object detection [5,6,7] has become a common way to extract information from visual data for construction applications, as it is more accurate and versatile than traditional machine vision techniques. To date, there have been numerous deep-learning-based automated solutions for visual construction monitoring in the areas of quality control [8,9,10], construction safety [11,12,13], and progress monitoring [14,15].

Although deep learning methods have been applied successfully for many image classification and object detection tasks, its applications to visual construction monitoring are still limited to tasks where the detected objects are distinctly different from background objects. For example, Wang et al. [14] proposed a precast wall installation monitoring solution by detecting isolated precast walls that are visible on the top level of a constructed building. Zheng et al. [15] developed a building progress monitoring system based on the detection of individual 3D prefabricated modules before they are installed and merged on the constructed building.

However, in many real-world visual progress monitoring tasks, the objects in the background may be similar to the targeted objects, which can therefore result in irrelevant detections. Additionally, the information within visual data is often incomplete or distorted due to sub-optimal camera angles caused by imaging constraints or the presence of occlusion and visual noise, which can result in missed detections. Furthermore, it may be hard to achieve robust detection if the targeted objects are depicted in large quantities, vary in scale, or contain little visual detail. 

**Figure 1:**
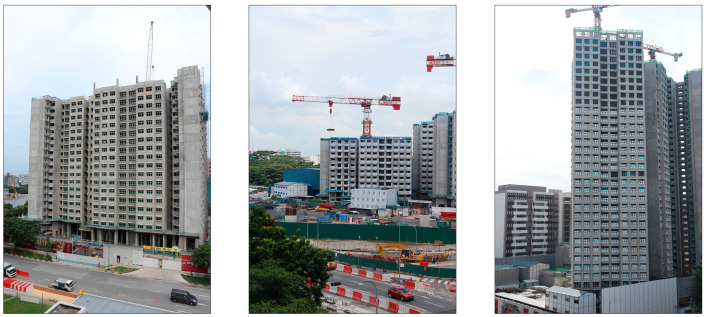
Images that depict PPVC buildings under construction.

In recent years, prefabricated prefinished volumetric construction (PPVC) has become increasingly prominent globally. PPVC refers to a type of modular construction where prefinished volumetric building modules are manufactured off site before being assembled on site to form a complete building structure [16]. Currently, PPVC projects can be found worldwide in countries such as the US, the UK, Australia, and China [17,18,19]. In countries like Singapore, PPVC has become a mandatory construction approach for select residential infrastructures [20]. Given the modular nature of PPVC, building construction progress monitoring for PPVC buildings can be conducted by quantifying assembled PPVC modules within images that depict the exterior of a constructed building, as shown in Figure 1. In the context of this paper, building construction refers to the assembly of PPVC modules. It was observed that most of the front-facing modules come with pre-installed window frames. As such, the quantification of assembled PPVC modules can be achieved by detecting and counting the number of windows on a constructed PPVC building. However, achieving robust window detection is challenging, as construction sites are visually complex and prone to occlusion, and windows from nearby infrastructure may also be detected. While several existing works have implemented object detection to monitor building construction [15,21], these methods did not consider measures to ensure that the detections used were robust.

Automating the progress monitoring process for PPVC buildings would allow for consistent and unbiased reporting. However, it is important to ensure the estimated progress is reliable. As such, this paper proposes a 2D window-based automated visual building construction progress monitoring (WAVBCPM) system for multi-storey PPVC building construction that uses the known structural layout of a constructed building to augment window detections made by an object segmentation model, with the following core functionalities:

- Elimination of window detections that are erroneous or irrelevant to progress monitoring of the target building by developing a filtering process, which consists of building mask filtering and arrangement of detected windows into columns.
- Prediction of missed window detections in the mid-section and near-ground regions of the constructed building via identification of anomalies within each window column.

Augmented window detections are subsequently used to estimate the modular assembly progress. The proposed method was tested based on real-world test cases where the test images depicting active PPVC construction sites around Singapore were taken by both the authors and the general public. The efficacy of WAVBCPM was assessed based on the similarity of the output progress estimates to manually determined ground truth progress and the time taken to complete progress estimation. The results show that WAVBCPM outperformed purely data-driven object detectors in building progress monitoring.

## 2. Related Work

Existing automated visual construction monitoring methods can be mainly segregated into point-cloud-based 3D methods and detection-based 2D methods. This section reviews both existing image-based 3D and 2D construction monitoring methods and assesses their applicability in monitoring progress for multi-storey PPVC buildings.

### 2.1. 3D Image-Based Solutions for Progress Monitoring

In progress monitoring, a common way of obtaining 3D point cloud data is photogrammetry, which reconstructs image data into point clouds [22] that are used to perform precise comparisons to 3D building information modelling (BIM) information [23,24]. This section discusses several existing photogrammetry-based works to evaluate the benefits and disadvantages of implementing photogrammetry for construction progress monitoring applications.

Omar et al. [25] used photogrammetry to obtain point clouds of concrete columns, which were then compared against the BIM model to determine construction progress. Although precise comparison could be made between the obtained point cloud and BIM data, it was mentioned that the image data collection could only be conducted during off-peak hours, as the implemented photogrammetric reconstruction can be affected by visual noise, dynamic occlusion, and lighting issues within the construction environment. In a proposed method for building construction progress monitoring by Bognot et al. [26], the implemented photogrammetric reconstruction of constructed buildings using datasets that contained between 391 and 783 images took between 31 and 55 h to complete. It is evident that photogrammetry is highly sensitive to environmental factors and is also time-consuming.

Based on the review of existing photogrammetry-based works, point clouds obtained using photogrammetry are found to be versatile and highly compatible with BIM data, which allows for precise progress monitoring. However, obtaining point clouds for large infrastructures comes at a higher cost compared to 2D data and is also extremely time-consuming to obtain and computation-heavy to process. Moreover, given the susceptibility of point clouds to dynamic noise, point cloud acquisition can only occur during off-peak hours when there is minimal movement within the environment. As PPVC building construction progress monitoring does not require the high precision offered by 3D techniques [27], implementing a 2D solution would therefore be more efficient.

### 2.2. 2D Object Detection for Progress Monitoring

In recent years, deep-learning-based object detectors have seen increased prominence in construction-based 2D object detection because they perform better than traditional methods at object detection tasks [5,6,28].

In the field of progress monitoring, the use of deep learning techniques was found in several works. Wei et al. [29] adapted a Mask R-CNN model into an instance segmentation model to measure progress for wall plastering based on the number of pixels within the plastering region as a percentage of the pixels in the wall. Rahimian et al. [21] incorporated the use of FuseNet to identify various target objects such as columns and beams through semantic segmentation of RGB-D data for the purpose of creating a virtual environment that is used for progress monitoring. A pretrained convolutional neural network (CNN) was also added in an object removal module to identify and remove unwanted objects such as humans, but these objects must be different from the target objects. Wang et al. [14] proposed a method for progress monitoring of precast wall installation using a finetuned Mask R-CNN model to detect precast walls before using the DeepSORT algorithm to track installation completeness. However, the progress monitoring in this work was only applicable to the top constructed storey of a building, where the precast walls were isolated and visible. As such, further information such as the constructed storey count had to be sourced externally to evaluate the progress of the constructed building. Zheng et al. [15] proposed the use of virtual prototyping to supplement training data for the transfer learning of a Mask-RCNN model for module detection in modular construction. Although it was mentioned that the findings of this work are applicable towards building construction progress monitoring, the implemented detection was targeted only at isolated modules before they were installed onto the constructed building. As such, the issues pertaining to high volume detection of PPVC modules were not considered. Furthermore, the focus of this work lies in dataset augmentation, and no algorithm was developed to apply the detections toward a statistical progress estimation for PPVC building construction. 

As construction sites are visually noisy, it is crucial to identify and overcome detection errors. This is seen in various construction-related object detection applications. Li and Li [30] used generative adversarial networks (GANs) to rectify inaccurate bounding boxes due to occlusion by predicting missing visual information in the context of worker detection. Chen et al. [31] eliminated background noise by blacking out regions in the input image that were irrelevant to the detection of cracks on buildings. Although the works by Wang et al. [14] and Zheng et al. [15] incorporated the use of 2D object detection to identify prefabricated components for progress monitoring, they did not propose any methods to overcome detection errors. In the work by Rahimian et al. [21], the proposed occlusion removal technique was ultimately still dependent on the detection robustness of the occlusion itself. 

As such, the existing 2D progress monitoring techniques are limited to tasks where the detected objects are distinctly different from background objects and are also unable to comprehensively consider the relevance and robustness of the detections to ensure that the detected visual information is accurate. As building features on multi-storey PPVC buildings tend to be visually repetitive, the detected building features can be analysed algorithmically instead to identify missed building features that were caused by occlusion. In addition, irrelevant and erroneous detections due to the surrounding environment could also adversely affect progress estimation and would therefore need to be addressed as well.

## 3. Methodology

In this section, the motivations and mechanisms of WAVBCPM are detailed. First, Section 3.1 explains the various difficulties of estimating the construction progress of multi-storey PPVC buildings using pure object detectors and discusses how WAVBCPM is designed to resolve these issues. Next, Section 3.2, Section 3.3 and Section 3.4 provide a detailed description for each module in WAVBCPM.

### 3.1. WAVBCPM Overview

**Figure 2:**
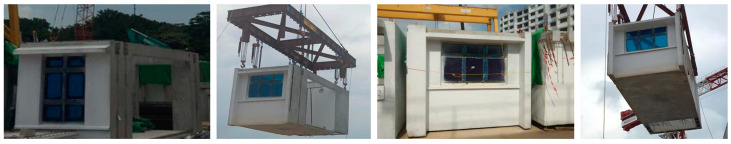
Images of PPVC modules taken from different angles.

PPVC assembly refers to a process in which prefabricated modules are assembled in a particular arrangement according to as-planned data. As shown in Figure 2, these prefabricated modules are commonly pre-installed with a window frame component on the exterior face of the module. As such, PPVC assembly progress can be estimated based on the detected number of window frames within visual data.

**Figure 3:**
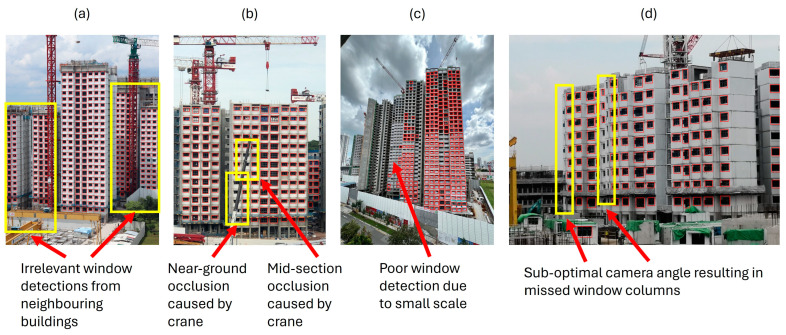
Examples of pure window detection output by a finetuned YOLOv5-S [32] object detection model. Issues identified within each image: (**a**) detection of irrelevant or erroneous windows; (**b**) heavy machinery, construction materials, and temporary constructs causing occlusion of windows; (**c**) missed windows during detection due to small scale; and (**d**) missed windows due to sub-optimal camera angles causing some window columns to be out of frame or occluded.

Transfer learning can be used to finetune a pretrained object detector for window detection based on a collated image dataset depicting PPVC buildings. However, simply detecting and counting the number of detected windows in an image using pure object detectors is not sufficient for conducting real-world progress monitoring of building construction, as there are various detection-related issues that can affect the estimated progress, as shown in Figure 3.

Firstly, some predicted windows might be irrelevant to building construction progress monitoring if they are from nearby infrastructures, unassembled PPVC modules on the construction site, or erroneous detections, as shown in Figure 3a. Secondly, construction sites are commonly littered with heavy machinery and construction materials. These objects often result in windows being occluded, as shown in Figure 3b. Some windows may also be missed during detection due to several constraints, such as distances and viewpoints of cameras. For example, small-scale windows are found in images depicting high-rise or wide buildings, as illustrated in Figure 3c, as capturing the whole building would require the camera to either be far away from the building or tilted at an angle, which causes windows that are further away from the camera to appear much smaller and hence harder to be detected. Additionally, the targeted windows may also vary in size according to the design as planned. Lastly, due to the positional constraints of cameras, the depiction of buildings within image data is occasionally sub-optimal, as part of the building might be out of frame, or the building itself could be occluding some windows, as shown in Figure 3d. 

**Figure 4:**
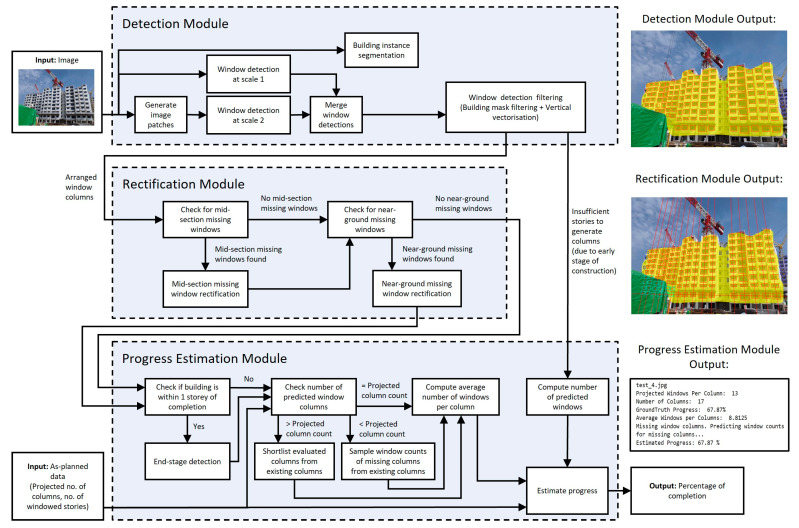
The architecture of WAVBCPM comprises the detection, rectification, and progress estimation modules. The detection module detects windows on the target building within an image and eliminates irrelevant detections. The rectification module accounts for missed window detections due to occlusion or poor detection. Lastly, the progress estimation module extracts relevant information from the processed window detections to estimate building construction progress.

Given these difficulties, WAVBCPM is proposed as a system that aims to improve the robustness of window detection while overcoming the issues of missing, irrelevant, or erroneous detections. The architecture of WAVBCPM comprises the detection module, rectification module, and progress estimation module, as shown in Figure 4. 

An overview that briefly describes the functionalities and motivations of each module is as follows:

(i) The detection module is responsible for localising windows on the target building within an input image. This is achieved by first conducting two-scale window detection, which is performed by extracting patches from the input image before conducting window detection on both the image and extracted patches. Output window detections are then concatenated together before duplicated detections are merged. The two-scale window detection approach is designed to improve window detection robustness by accounting for targeted windows at smaller scales. Next, the window detections are filtered using a building mask obtained from an instance segmentation model and arranged into columns via a vertical vectorisation algorithm to eliminate irrelevant and erroneous detections, such as in Figure 3a. The implemented filtering mechanisms are designed to mimic how humans are able to identify and focus on the monitored building based on its distinct features and repetitive construction patterns during manual progress monitoring. If no window columns were output, building construction is still at an early stage. In such cases, the number of detected windows is directly used to estimate building construction progress.
(ii) As building construction is a ground-up process, a human would count assembled modules in the lower and mid-section regions of the constructed building even if they are occluded. This is reproduced algorithmically by sending the arranged window columns into the rectification module, which identifies and rectifies missed window detections due to occlusion or poor detection, as illustrated in Figure 3b. Missed window detections are classified as either mid-section or near-ground to be resolved separately. Note that missed window detections on the top-most storey of a constructed PPVC building were not considered for rectification because the top-most storey could still be under construction. Furthermore, occlusions that block the window components of PPVC modules are usually found only in the lower to middle regions of the constructed PPVC building. For the case of mid-section missing windows, erroneous regions are identified by significant gaps between windows in each column. Each erroneous gap is defined by the detected windows above and below it. For the case of near-ground missing windows, there are no detected windows below the erroneous region. To account for near-ground missing windows, a horizontal line vector is estimated to localise the lowest windowed storey of the constructed building. Using the obtained line vector, near-ground missing windows are identified and rectified based on the location of the bottom-most windows in each column.
(iii) Lastly, the progress estimation module extracts the average number of windows per column from the rectified window columns to predict building construction progress. If building construction is near completion, end-stage detection is conducted to bypass any residual detection errors or occlusion. This is achieved by identifying the roof components that are installed along with the top-most PPVC modules on a completed building.

To ensure that there are no extra predicted windows or missing window columns, the output number of window columns is compared against the as-planned window column count, which can be obtained from BIM. If there are too many predicted window columns, the columns are assessed and shortlisted based on the deviations in their window counts from the median window count. Conversely, if there are missing window columns, the window counts of existing output columns are used to form a probability distribution detailing the frequency of occurrence for each unique window count. The obtained distribution is then sampled probabilistically to predict the window counts of missing columns.

### 3.2. Detection Module

The detection module consists of two-scale window detection and window detection filtering. Section 3.2.1 and Section 3.2.2 provide detailed descriptions of these two processes.

#### 3.2.1. Two-Scale Window Detection

**Figure 5:**
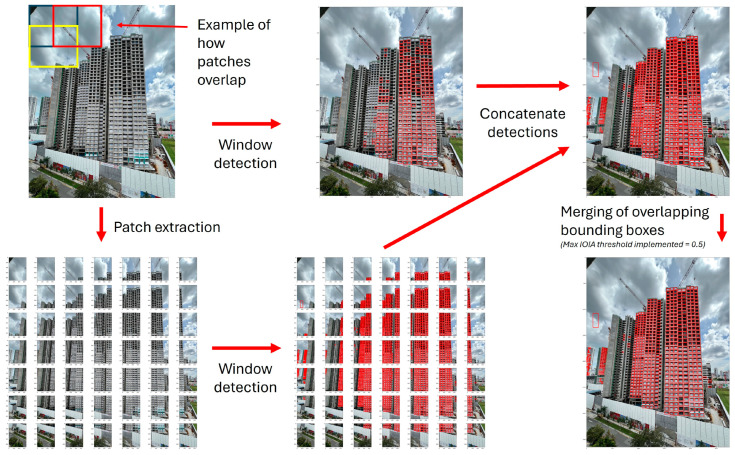
Two-scale window detection pipeline.

As the windows captured in images can be small and vary in size, WAVBCPM conducts window detection at two scales to improve window detection sensitivity. This is achieved by extracting patches from the input image, which are then sent for window detection alongside the original input image, as shown in Figure 5. The patch extraction process first divides the input image into n-by-n overlapping patches. Each patch has size [2x/(n + 1), 2y/(n + 1)], with x and y representing the dimensions of the original image. Patch parameter n serves as a scaling tool to ensure that the targeted features are clearly visible and distinct, where a larger n value increases the magnification of details in each patch at the expense of computing time. In the experiments, n was set by default to 7 to accommodate window detection for extremely small windows. Additionally, the same number of patches were selected along the horizontal and vertical dimensions to preserve the dimension ratio of the original image to prevent the patches from unintended warping.

Next, window detection was conducted for all extracted patches along with the original input image. All window detections were selected with an empirically optimised confidence threshold. Following window detection at two scales, the extracted patches were then stitched back together. Bounding box coordinates of window detections in each patch were re-localised with respect to the original image. Window detections from both the patches and the original image were concatenated together. Some windows may have experienced multiple detections as they appeared in more than one extracted patch. Notably, the overlap between patches allowed for all bounding box detections that were predicted for the same window to share some degree of intersection. To evaluate this degree of intersection, the maximum intersection-over-individual-area (IOIA) was calculated based on the areas of the boxes, as follows:

(1) $$\max IOIA=\frac{box1_{area}\cap box2_{area}}{min(box1_{area}\;,\;\;box2_{area})\;}$$

Bounding box pairs with a max IOIA value that exceeds a predefined merging threshold of 0.5 are recursively merged until all remaining bounding box pairs do not meet the merging requirement. 

Two-scale window detection for WAVBCPM can be implemented using any finetuned object detector as a backbone. In the proposed method, a YOLOv5-S object detection model was finetuned for window detection. A total of 120 images featuring the PPVC assembly process in various stages, angles, and distances were collated from 15 construction sites around Singapore and BTOHq [33], a website with crowd-sourced images of PPVC construction in Singapore. In total, 9386 bounding boxes were manually labelled for windows found in the collated images using LabelImg [34]. The compiled images were then split into training and validation datasets containing 100 and 20 images, respectively, for the finetuning and optimisation of window detection. The weights of the implemented YOLOv5-S were finetuned via transfer learning using the training dataset, and the validation dataset was used to assess the performance of the models at the end of each finetuning epoch. The learning ended when the attained mean average precision at an IOU threshold of 0.5 (mAP@0.5) stagnated. The window detector was able to achieve a high mAP@0.5 of 0.96. The implemented confidence threshold for the window detector was also optimised. This was achieved by thresholding the output window predictions for images in the validation dataset using a range of confidence thresholds between 0 and 1 with a step size of 0.01 and comparing the output window detections to the defined ground truth bounding boxes. A predicted bounding box was determined to be accurate if it shared an IOU of at least 0.50 with a ground truth bounding box. By computing the true positives (TPs), false positives (FPs), and false negatives (FNs) at each confidence threshold, an F1 score could be derived. Based on comparison between the F1 scores attained by different confidence thresholds, it was empirically found that conducting window detection at a confidence threshold of 0.75 was optimal.

#### 3.2.2. Window Detection Filtering

**Figure 6:**
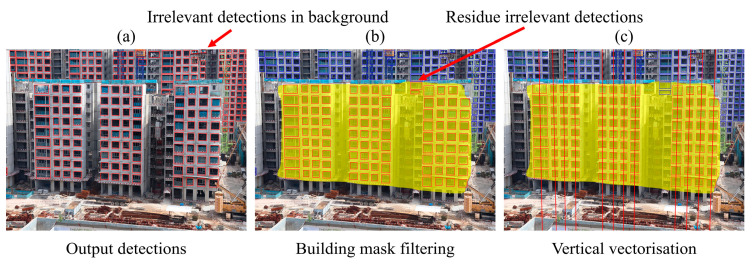
(**a**) Initial set of detected windows before filtering. Detections (**b**) after building mask filtering and (**c**) vertical vectorisation.

Although two-scale window detection is capable of detecting smaller windows and even windows of different sizes, some detected windows may be irrelevant to progress estimation. They mainly consist of windows detected on nearby infrastructure and miscellaneous objects on the construction site. As such, building mask filtering and window column vectorisation are implemented to eliminate irrelevant detections, as shown in Figure 6. 

Building mask filtering is first implemented to target window detections found on the monitored building. A visual mask of the target PPVC building is predicted using a finetuned Cascade Mask R-CNN (SWIN-T) instance segmentation model to serve as a region of interest that identifies relevant detections. As WAVBCPM targets only one building per image, only the building segment with the highest confidence that exceeds an empirically optimised confidence threshold is selected. Any window detection with a bounding box that has less than two corners in contact with the building mask is filtered from the pool of detections. Note that the collated dataset, finetuning, and confidence threshold optimisation procedures discussed in Section 3.2.1 were similarly used to adapt the Cascade Mask R-CNN (SWIN-T) for building segmentation. However, there are several differences. Firstly, the 120 buildings depicted in the collated dataset were manually segmented using Labelme [35] to serve as a ground truth in place of ground truth bounding boxes. Secondly, a higher IOU requirement of 0.70 was used when evaluating predicted building segments, as the predicted building mask needs to cover the target building extensively to be deemed accurate. The finetuned Cascade Mask R-CNN (SWIN-T) was able to achieve a high mAP@0.5 of 0.986. It was also found from the confidence threshold optimisation that conducting building segmentation at a confidence threshold of 0.91 is optimal.

Next, the remaining detection bounding boxes within the building mask were arranged into columns via vertical vectorisation to eliminate residual irrelevant detections and facilitate subsequent processing measures. Detections that did not fit into the predicted columns were deemed erroneous and were eliminated. A pseudocode for the window detection filtering process is presented in Algorithm 1.

Vertical vectorisation begins by sorting bounding boxes according to their centre x-coordinates. For each ranked box *b_i_*, eight bounding boxes {*b_i_*_1_, *b_i_*_2_ *… b_in_*} that ranks closest to *b_i_* are shortlisted. The Euclidean distances {*d_i_*_1_, *d_i_*_2_ *… d_in_*} and y-coordinate differences {*y_i_*_1_, *y_i_*_2_ *… y_in_*} between the centre coordinates of each shortlisted box and *b_i_* are calculated to identify the boxes *b_i-top_* and *b_i-bot_*, which are directly above and below *b_i_*. If either *b_i-top_* or *b_i-bot_* cannot be obtained, *b_i_* is determined to be a detected window on the top-most or bottom-most windowed storey of the target building and thus is omitted from consideration. A line vector *v_i_* is estimated by minimising L2 distance as follows:

(2) $$\sum_{k=1}^{3}\sqrt{{\left(x_{ik}-x_{line_{ik}}\right)}^{2}+{\left(y_{ik}-y_{line_{ik}}\right)}^{2}}$$

where *x_ik_*, *y_ik_* (*k* = 1, 2, 3) denotes the centre coordinates of *b_i_*, *b_i-top_*, and *b_i-bot_*, while *x_lineik_* and *y_lineik_* represent the closest point on vi to the centre coordinates of *b_i_*, *b_i-top_*, and *b_i-bot_*. Bounding boxes that are intersected by *v_i_* are collated and denoted as *c_i_*. *v’_i_* is then obtained from the centre coordinates of bounding boxes in *c_i_* by minimising L1 distance as follows:

(3) $$\sum_{k=1}^{n}\;\left|{x\prime }_{ik}-{x\prime }_{line_{ik}}\right|+\left|{y\prime }_{ik}-{y\prime }_{line_{ik}}\right|$$

where *x’_ik_* and *y’_ik_* (*k* = 1, 2, 3,…, n) denote the centre coordinates of bounding boxes in *c_i_*, while *x’_ik_* and *y’_ik_* represent the closest point on *v’_i_* to the centre coordinates of the bounding boxes in *c_i_*. *c’_i_* is then computed using *v’_i_* to eliminate outlier bounding boxes in ci. Each *c’_i_* represents a potential column of windows. However, if the derived gradient *g’_i_* of *v’_i_* is greater than a predefined gradient threshold, then *v’_i_* is omitted from consideration, as the output window columns should visually be near-vertical. Notably, if the camera is positioned close to the constructed building, this may result in predicted column vectors with gradients that deviate further from the vertical axis. As such, the gradient threshold was set by default to 20° to account for sub-optimal camera angles whilst still being able to effectively eliminate stray column vectors. 

Next, a merging operation is conducted to eliminate duplicate, subset, and stray window columns. Each column of windows *c_a_* in *C* is compared to every other window column *c_b_*. If *c_a_* is found to be a duplicate or subset of *c_b_*, then *c_a_*, its corresponding vector *v_a_*, and derived gradient *g_a_* are eliminated. At the end of the merging operation, window columns that were not merged are eliminated.

Following the merging operation, the predicted vectors are checked for intersection. Each intersection occurrence r from the identified list of intersection clusters *R* contains a set of intersecting vectors {*v_a_*, *v_b_ … v_n_*}. If an intersection occurs between two or more vectors, the intersecting vector with the greatest difference in gradient when compared against the nearest non-intersecting vector is eliminated. This process occurs recursively, with one vector removed per intersection occurrence until there are no intersections within the output vectors.
**Algorithm 1.** Window detection filtering pseudocode
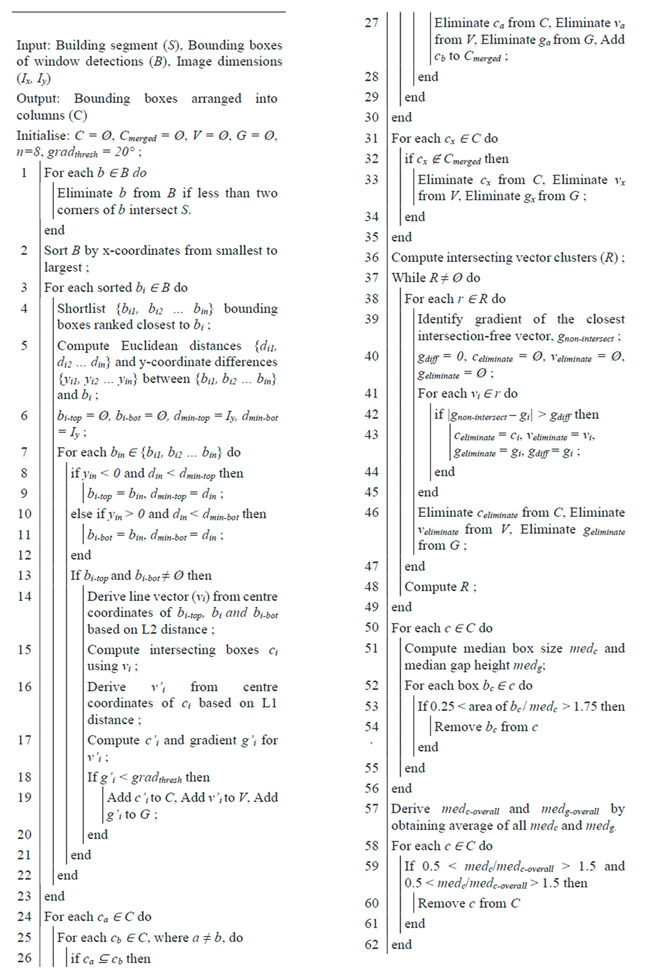



Note that vertical vectorisation requires a minimum of two predicted vectors to localise a column of windows. As such, its implementation would require the building to be at least four storeys high given that three windows are required to predict a vector. If this requirement is not fulfilled, the rectification and progress estimation modules are skipped. The total number of detected windows is instead given as a percentage of the projected window count for the depicted building face, as shown in Figure 4.

**Figure 7:**
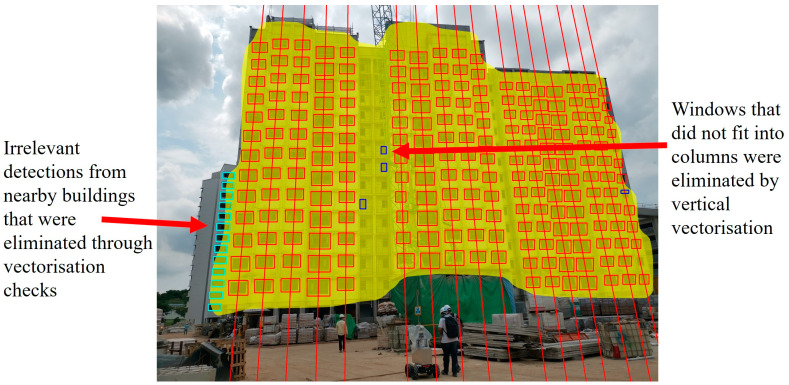
Output window columns predicted by vertical vectorisation are annotated by line vectors. Through vertical vectorisation and the implemented vectorisation checks, erroneous and irrelevant window detections that passed through building mask filtering could be identified and removed.

Lastly, the predicted building mask acquired through segmentation may occasionally be erroneous due to visual noise or unattainable if none of the building segments pass the confidence thresholding criterion. To account for potential building segmentation errors and further minimise detection errors within the predicted window columns, each window column is screened for bounding boxes that possess extreme differences in size to the other boxes in the column. Additionally, a window column is also determined to be erroneous if it possesses both bounding boxes and gaps between bounding boxes that are significantly smaller than other columns. Note that the implemented checks may be deactivated if extreme differences in window size are intended as part of the design of the constructed building. Details on the implemented measures are as follows:

(i) Vectorisation check 1: A median box size is derived for each column by arranging the areas of its bounding boxes in ascending order and selecting the area value in the middle. If there are an even number of predicted window columns, then an average of the two areas in the middle is used. Bounding boxes within the column that deviate from the derived median box size by more than 75% are eliminated.
(ii) Vectorisation check 2: The median box size and the median gap height from each column are used to derive an overall median box and gap height in a similar way to measure 1. If both the median box and gap height for a predicted column differ by more than 50% from the derived overall median box and gap height, the predicted window column is likely detected from a nearby building in the background and is thus eliminated. Figure 7 illustrates a scenario where irrelevant window detections that were unable to be filtered by the predicted building mask were eliminated using the conducted vectorisation checks.

### 3.3. Rectification Module

The columns of windows output from the detection module are sent into the rectification module to be assessed for missing windows due to missed detections or occlusion. Section 3.3.1 and Section 3.3.2 detail the steps taken to identify and rectify mid-section and near-ground missing windows.

#### 3.3.1. Mid-Section Missing Window Rectification

Missing windows within the mid-section of a column are identified by evaluating the gaps between the centre coordinates of detected windows. A gap would require rectification if it is determined to be abnormally large. To identify erroneous gaps, K-means (K = 2) clustering is used to cluster the heights of gaps within each column in order to classify the gaps into two groups: erroneous and non-erroneous. Note that the heights of gaps are squared to amplify the presence of outliers prior to clustering. If the subsequent mean gap heights of the two clusters differ by more than 100%, the cluster with the larger mean gap height is determined to contain gaps that require rectification.

**Figure 8:**
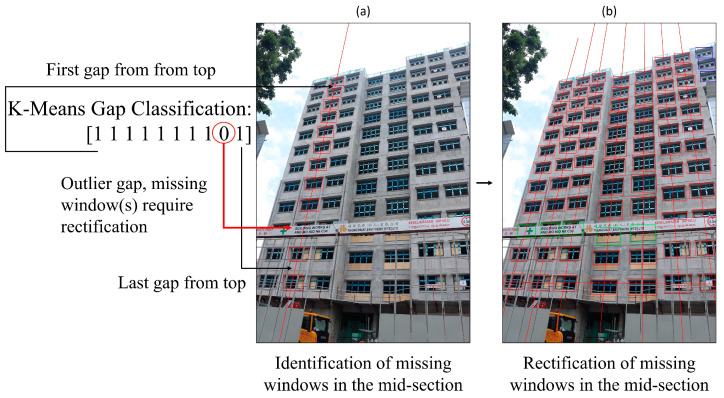
Mid-section (**a**) identification and (**b**) rectification of missing windows. Bounding boxes that were added during rectification are annotated in green.

Each erroneous gap is rectified by recursive addition of bounding boxes to its top-most region until an added bounding box intersects the bottom bounding box that defines the erroneous gap. The dimensions of added bounding boxes are aggregated by averaging the dimensions of four bounding boxes within the evaluated column closest to the erroneous gap. The added spacing between boxes is approximated from two non-erroneous gaps within the column closest to the erroneous gap. A visual representation of mid-section missing window rectification is shown in Figure 8.

#### 3.3.2. Near-Ground Missing Window Rectification

Near-ground missing windows are identified by evaluating the position of the bottom-most window within each column with respect to the lowest windowed storey, which is localised by a horizontal line vector. A column is determined to require near-ground rectification if its bottom-most window does not intersect the horizontal line vector. 

**Figure 9:**
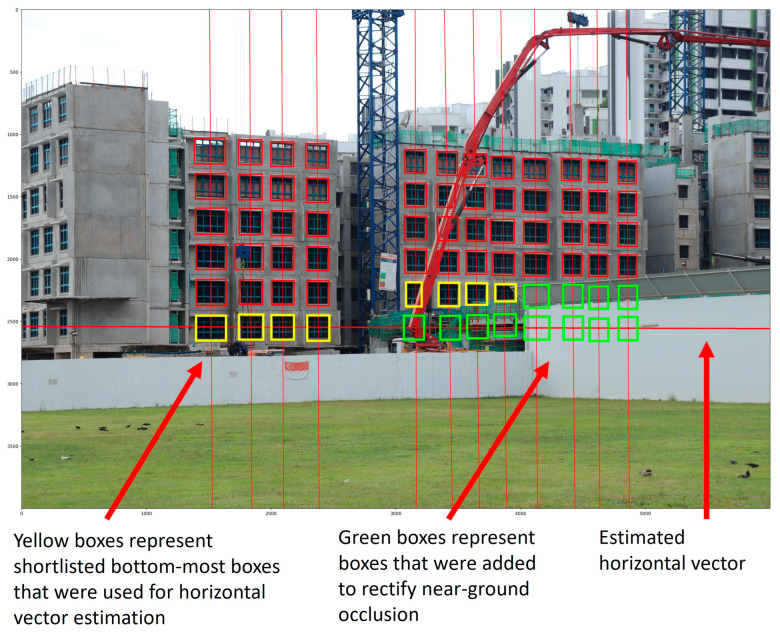
A visual representation of near-ground missing window rectification. Yellow boxes represent the shortlisted boxes used to estimate the horizontal line vector. Green boxes represent added boxes used to rectify near-ground missing windows. Red boxes represent window predictions that were unused for the rectification step.

L1 distance is first used to estimate the horizontal line vector based on the shortlisted centre coordinates of bottom-most windows from columns with a window count that deviates from the mode window count by at most one. Notably, if the unique window counts from the shortlisted columns are evenly distributed, such as in Figure 9, it may cause the estimated horizontal line vector to be slanted. Therefore, shortlisted columns with a lower window count are removed from consideration in such cases. In addition, if three or more shortlisted bottom-most boxes fall beneath the estimated horizontal line vector, this implies that the estimated vector is not yet localising the lowest windowed storey. In such scenarios, the horizontal line vector is re-estimated using boxes beneath the current estimated line vector. 

Columns with identified near-ground missing windows are rectified by recursive addition of bounding boxes to the bottom of the column until its bottom-most bounding box intersects the estimated horizontal line vector. Additionally, the window count of the rectified columns should not exceed the mode window count. The dimensions and spacings of the added boxes are approximated from the four bottom-most boxes and the two bottom-most gaps within the rectified column. The vector estimation and rectification processes are then repeated until no further boxes are added. An example of near-ground rectification is visualised in Figure 9.

### 3.4. Progress Estimation Module

The progress estimation module evaluates the output window columns and converts the obtained visual information into statistical data. This is achieved through the end-stage detection, window columns check, and progress estimation processes described in Section 3.4.1 and Section 3.4.2.

#### 3.4.1. End-Stage Detection

If PPVC assembly is within one storey of completion, a check for building construction completion is conducted by identifying the roof components that are typically installed along with PPVC modules on the top-most storey of a constructed building.

**Figure 10:**
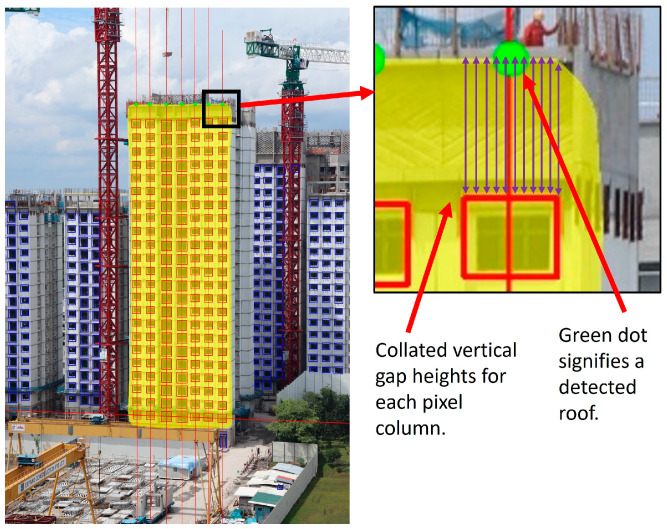
End-stage detection conducted on output columns of windows represented by red boxes. Blue boxes represent window predictions that were filtered by earlier procedures. A window column is determined to be complete if a green circle is annotated above it.

For each window column, the left and right x-coordinates of the top-most bounding box serve as a bound for the evaluated region. If a roof component is installed above the completed window column, there would be a significant gap between the top edge of the building mask and the top-most window. This gap is aggregated by averaging the vertical pixel differences between the top edge of the building mask and the top edge of the top-most bounding box within the bounded region. If the derived average gap height is significantly large, the roof component is considered to be installed. The corresponding window column would therefore possess a window count that is equivalent to the completed storey count regardless of the number of window predictions it contains. Note that the actual roof and window dimensions can be converted to a ratio to evaluate the aforementioned averaged difference. For generic implementation purposes, the evaluated gap is considered significant if it has a height that is at least half the height of the top-most bounding box. An example of end-stage detection is shown in Figure 10.

#### 3.4.2. Window Columns Check and Storey-Based PPVC Progress Estimation

Before the building construction progress is calculated, the number of output window columns is checked against as-planned data to determine if there are missing or excess window columns predicted. Additionally, any column with a window count that exceeds the number of projected storeys is also removed, as it is likely affected by prediction error.

For the case of excess window columns, a median window count is first derived by arranging the window counts of existing window columns in ascending order and then choosing the window count in the middle. If there is an even number of predicted window columns, then an average of the two window counts in the middle is used. The deviation of the window count of each column from the median window count is then calculated. Recursively, the window column that possesses the window count with the largest deviation from the median window count is removed from consideration, until the number of remaining window columns matches the number of as-planned window columns. If the largest deviation for an iteration is 0, it implies that the remaining window columns all have the same window counts. As such, a random window column will be removed, as it will not affect the output average window count.

For the case of missing window columns, the frequency of occurrence for each unique window count is first collated from the existing window columns. The probability of a missing column having a particular unique window count is calculated by taking the unique frequency of occurrence of the window count as a fraction of the total number of predicted window columns. The product of each obtained probability and the number of missing columns is then obtained and rounded to an integer to estimate the number of occurrences for its corresponding window count within the missing window columns. The predicted missing window counts are then concatenated with the existing window counts for progress estimation.

**Figure 11:**
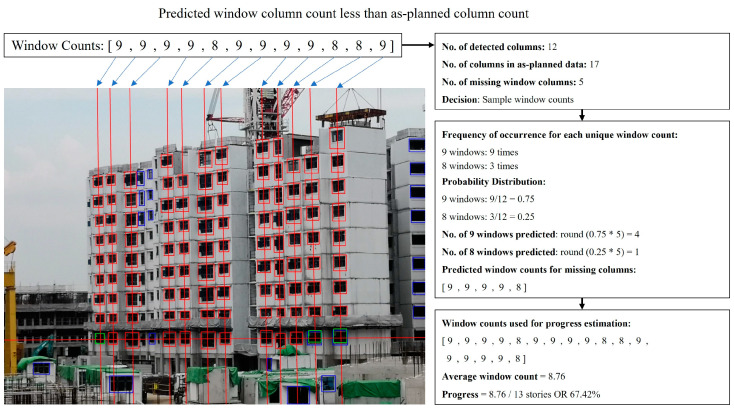
Output window columns represented by red and green boxes are assessed. Blue boxes represent window predictions that were filtered by earlier procedures. For this given image, there are several missing window columns.

Lastly, the average window count per column is compared against the projected number of windowed storeys in as-planned data to estimate building construction progress. Figure 11 illustrates the steps taken to assess and estimate progress for a given image.

## 4. Evaluating the Progress Estimation of WAVBCPM

This section assesses the PPVC assembly progress estimation capabilities of WAVBCPM by comparing its detection and progress estimation capabilities to various pure object detector (POD) models comprised of YOLOv5 and Cascade Mask R-CNN (Swin) variants based on similarity to a set of manually determined ground truth detections and progress. Progress estimation for the POD approach is conducted by comparing the number of detected windows to the as-planned number of windows on the depicted building face in each image. Building construction is assumed to be complete if the estimated progress exceeds 100%. Note that the POD models were finetuned and optimised using the collated image dataset and procedures discussed in Section 3.2.1, which was used to finetune the YOLOv5-S model that was incorporated as the window detection backbone of WAVBCPM.

### 4.1. Experiment Setup

**Figure 12:**
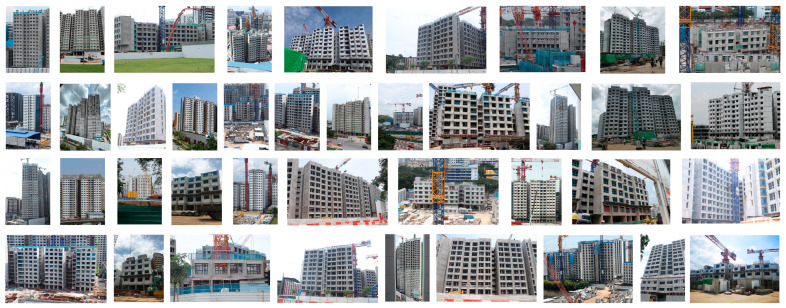
Images from test set A that were taken by the authors from construction sites around Singapore.

Two testing datasets, test sets A and B with 40 and 60 images, respectively, were used to investigate the performance of WAVBCPM. Test set A, which comprises 40 images taken from 17 construction sites, features images that were taken by the authors as shown in Figure 12. Test set B, which comprises 60 images taken from 21 construction sites, was sourced online from BTOHq [33]. The images in test set B were taken by the public without any knowledge of machine learning and the proposed algorithm. Note that the images in test set B are not presented in this paper due to copyright issues.

Conventional object detection evaluation metrics, such as TP, FP, and FN counts, are unable to comprehensively assess the efficacy of WAVBCPM. This is because WAVBCPM can account for fully occluded windows, which cannot be manually labelled in the test images. Moreover, WAVBCPM also uses other features, such as an assembled roof or window column information, to improve on the estimated progress. As such, assessing the final output progress would be a better measure to evaluate WAVBCPM because it can also reflect detection-independent improvements. Absolute deviation from the defined ground truth progress percentage was used as a metric to evaluate the estimated progress as follows:

(4) $$Absolute\;deviation=\left|a-b\right|$$

where *a* and *b* denote the compared progress percentages given that the estimated progress percentage can be either higher or lower than the ground truth progress percentage. For comparative purposes, the TP, FP, and FN detection counts for both test sets were also collected for both WAVBCPM and POD models. However, the window detections output by the automated methods were manually screened. If the window detections for fully occluded windows were correctly predicted, they were manually re-classified from FP to TP. 

### 4.2. Results

**Figure 13:**
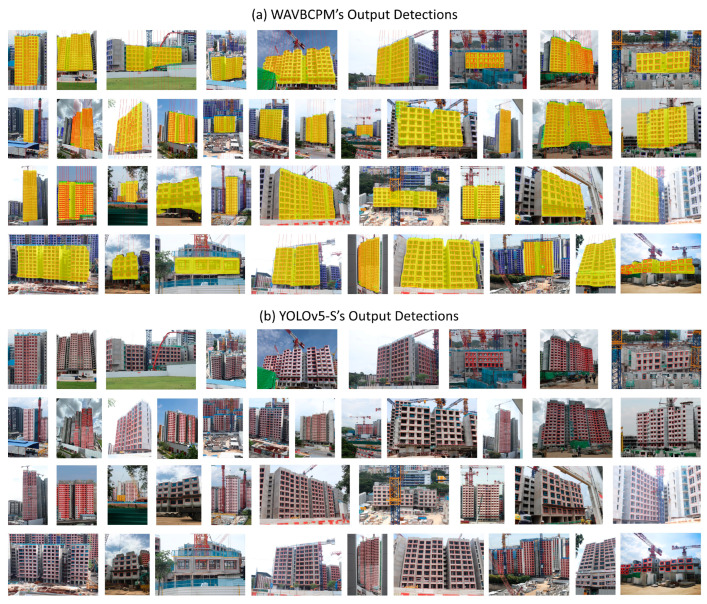
Detection outputs of (**a**) WAVBCPM and (**b**) POD (YOLOv5-S) for test set A.

Table 1 shows the screened TP, FP, and FN detection counts for test sets A and B. The efficacy of each automated method was assessed based on the maximum and mean absolute deviations between manually determined ground truth progress and the estimated progress, as shown in Table 2. In addition, the minimum, maximum, and average time taken to process the images were also recorded to compare the rate of computation between each method. Apart from comparing the estimated progress, the detections output by WAVBCPM and the POD (YOLOv5-S) are provided in Figure 13 to visualise differences in output detections.

### 4.3. Discussion

Based on the statistical data in Table 2, the progress estimated by WAVBCPM was almost identical to ground truth progress, as evident from the achieved mean absolute deviations of 0.14% and 0.26% for test sets A and B. In comparison, the POD models achieved significantly worse mean absolute deviations of 9.69% and 8.56%, respectively. In addition, the worst output progress estimated by WAVBCPM resulted in 2.27% and 2.60% maximum absolute deviations from the ground truth for test sets A and B. In contrast, the POD models had drastically higher maximum absolute deviations recorded at 56.25% and 58.34%. The obtained statistics are further supported by Table 1, which shows that the window detection performance of POD models had lower TP counts and significantly higher FP and FN counts in comparison to WAVBCPM. 

**Figure 14:**
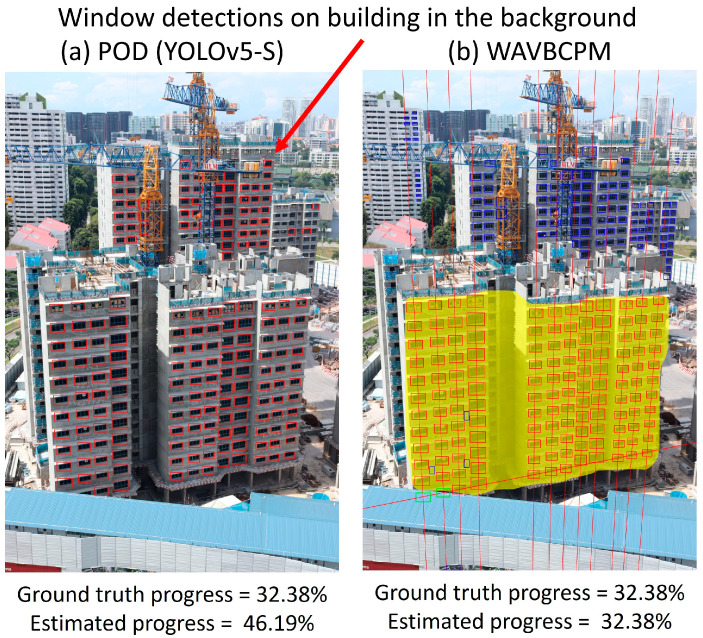
The POD (YOLOv5-S) model encountered significant errors when estimating progress for images with infrastructure-dense backgrounds due to many irrelevant detections (**a**). Conversely, WAVBCPM was shown to be able to pinpoint the target building and evaluate its progress accurately (**b**).

Analysis of the visual outputs from the POD (YOLOv5-S) model in Figure 13b also reveals that the significant inaccuracies in progress estimations by the POD model were primarily caused by irrelevant detections, especially when the image background was visually noisy. If there were many nearby buildings in the background, such as in Figure 14, the POD model could overestimate significantly due to window detections from nearby buildings. This observation surfaces a need to identify a target area for evaluation when implementing vision-based automation in infrastructure-dense environments.

**Figure 15:**
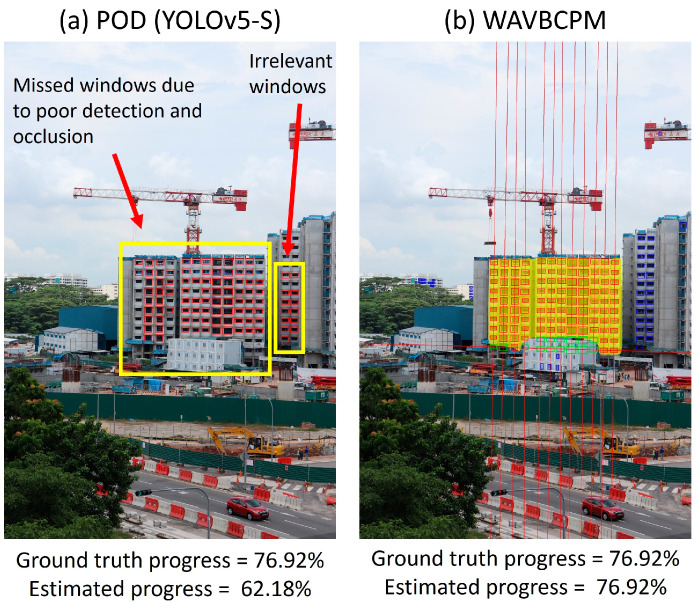
Missed and irrelevant windows can occur concurrently and offset each other during progress estimation by POD (**a**). In contrast, detection errors were rectified prior to progress estimation by WAVBCPM (**b**).

Moreover, there were also instances in the visual outputs illustrated in Figure 13b, where multiple types of window prediction error concurrently occurred for the POD model, such as in Figure 15a. This caused the progress estimated by POD models to be unreliable because irrelevant or erroneous window detections can offset missed or occluded windows. In contrast, WAVBCPM was able to rectify the detection errors prior to progress estimation, as illustrated in Figure 15b. 

Lastly, WAVBCPM was observably slower than the POD models. This was expected, given that WAVBCPM performs considerably more detections and data computations per image. In addition, there was also a significant difference between the minimum and maximum time taken by WAVBCPM to process the window detections and estimate progress, as the number of targeted windows may vary depending on building design and stage of construction.

Based on the results obtained from the conducted experiment, the application of pure object detection was found to be insufficient for PPVC assembly progress estimation because detected windows need to be correctly interpreted and irrelevant or erroneous window detections need to be filtered. The results showed that WAVBCPM is a viable automated building construction progress monitoring solution for PPVC buildings that works well even in building-dense environments. Furthermore, the empirical results obtained by WAVBCPM for test sets A and B were similar, which indicates that WAVBCPM does not require skilled labour to implement, as its accuracy was not compromised when the input images were taken by individuals with no precursor knowledge of the algorithm.

## 5. Conclusions

This paper presents a novel 2D building construction progress monitoring solution for PPVC called WAVBCPM. It consists of three modules designed to optimise window prediction for progress monitoring in real-world constriction scenarios, eliminate inaccuracies in detections, and accurately estimate PPVC assembly progress. Experimental studies conducted on 38 actual PPVC construction sites demonstrated the feasibility and accuracy of WAVBCPM in addressing real-world challenges.

From the experimental studies conducted, it was found that the progress estimated by WAVBCPM was highly accurate, as evident from the achieved mean absolute deviations of 0.14% and 0.26% for the two test sets. In contrast, the implemented POD models had a much larger margin of error in their progress estimations, with best mean absolute deviations of 9.69% and 8.56%. Additionally, there were also extreme instances of progress overestimation observed for the POD models, which did not occur with WAVBCPM. Although POD performance can be further improved by optimising the implemented detection model, externalities such as occlusions and irrelevant detections require specific considerations to be resolved to ensure accurately monitored construction progress. Based on the obtained test results, WAVBCPM was shown to be capable of identifying relevant and useful predictions before effectively translating the obtained visual data into a statistical output. Nevertheless, the current work focuses on PPVC buildings where external windows were visible and directly related to the structural construction progress. In other non-PPVC scenarios, such as when external windows are not visible during construction or when monitoring progress inside the building, future work could focus on training the object detection module in WAVBCPM to identify additional interior and exterior visual building features. The processing algorithm should also be adapted to meet the specific requirements of each scenario.

## Figures and Tables

**Table 1:** Screened TP, FP, and FN counts of test sets A and B for various automated window detection methods.

Test set A (40 images)			
Method	TP	FP	FN
POD (YOLOv5-S)	4709	1119	530
POD (YOLOv5-M)	4765	1227	474
POD (YOLOv5-L)	4771	1168	468
POD (Cascade Mask R-CNN (Swin-T)	4110	594	1129
POD (Cascade Mask R-CNN (Swin-S)	3990	500	1249
POD (Cascade Mask R-CNN (Swin-B)	4240	660	999
WAVBCPM	5180	101	59
Test set B (60 images)			
Method	TP	FP	FN
POD (YOLOv5-S)	5134	1011	452
POD (YOLOv5-M)	5152	1041	434
POD (YOLOv5-L)	5107	1058	479
POD (Cascade Mask R-CNN (Swin-T)	4791	762	795
POD (Cascade Mask R-CNN (Swin-S)	4722	667	864
POD (Cascade Mask R-CNN (Swin-B)	4872	833	714
WAVBCPM	5504	226	82

**Table 2:** Maximum and mean absolute deviation between manually determined ground truth progress and the progress estimations of POD models/WAVBCPM for test sets A and B. The minimum, maximum and average time taken to process a test image were also recorded.

Test set A (40 images)					
Method	Absolute Deviation		Time Taken Per Image		
	Max (%)	Mean (%)	Min (s)	Max (s)	Mean (s)
POD (YOLOv5-S)	56.25	10.07	0.03	0.54	0.33
POD (YOLOv5-M)	56.25	9.69	0.04	0.55	0.33
POD (YOLOv5-L)	56.25	9.74	0.04	0.55	0.34
POD (Cascade Mask R-CNN (Swin-T)	65.99	13.20	0.13	3.00	0.65
POD (Cascade Mask R-CNN (Swin-S)	65.99	13.20	0.15	2.78	0.65
POD (Cascade Mask R-CNN (Swin-B)	61.95	13.02	0.18	3.04	0.78
WAVBCPM	2.27	0.14	1.16	15.19	6.86
Test set B (60 images)					
Method	Absolute Deviation		Time Taken Per Image		
	Max (%)	Mean (%)	Min (s)	Max (%)	Mean (%)
POD (YOLOv5-S)	58.34	9.08	0.02	0.14	0.07
POD (YOLOv5-M)	63.89	8.56	0.02	0.17	0.08
POD (YOLOv5-L)	72.22	9.23	0.02	0.15	0.08
POD (Cascade Mask R-CNN (Swin-T)	77.78	11.02	0.11	0.81	0.24
POD (Cascade Mask R-CNN (Swin-S)	62.5	10.78	0.13	0.8	0.26
POD (Cascade Mask R-CNN (Swin-B)	77.78	10.28	0.14	0.82	0.29
WAVBCPM	2.60	0.26	0.78	8.74	2.42

## Data Availability

Data are contained within the article.
